# Infant feeding method and special educational need in 191,745 Scottish schoolchildren: A national, population cohort study

**DOI:** 10.1371/journal.pmed.1004191

**Published:** 2023-04-06

**Authors:** Lisa J. Adams, Jill P. Pell, Daniel F. Mackay, David Clark, Albert King, Michael Fleming

**Affiliations:** 1 School of Health and Wellbeing, University of Glasgow, Glasgow, United Kingdom; 2 Public Health Scotland, Edinburgh, United Kingdom; 3 ScotXed, Scottish Government, Edinburgh, United Kingdom

## Abstract

**Background:**

While special educational needs (SEN) are increasingly recorded among schoolchildren, infant breastfeeding has been associated with reduced incidence of childhood physical and mental health problems. This study investigated relationships between infant feeding method and risk of all-cause and cause-specific SEN.

**Methods and findings:**

A population cohort of schoolchildren in Scotland was constructed by linking together health (maternity, birth, and health visitor records) and education (annual school pupil census) databases. Inclusion was restricted to singleton children, born in Scotland from 2004 onwards with available breastfeeding data and who attended local authority mainstream or special schools between 2009 and 2013. Generalised estimating equation models with a binomial distribution and logit link function investigated associations between infant feeding method at 6 to 8 weeks and all-cause and cause-specific SEN, adjusting for sociodemographic and maternity factors.

Of 191,745 children meeting inclusion criteria, 126,907 (66.2%) were formula-fed, 48,473 (25.3%) exclusively breastfed, and 16,365 (8.5%) mixed-fed. Overall, 23,141 (12.1%) children required SEN. Compared with formula feeding, mixed feeding and exclusive breastfeeding, respectively, were associated with decreased all-cause SEN (OR 0.90, 95% CI [0.84,0.95], *p* < 0.001 and 0.78, [0.75,0.82], *p* < 0.001), and SEN attributed to learning disabilities (0.75, [0.65,0.87], *p* < 0.001 and 0.66, [0.59,0.74], *p* < 0.001), and learning difficulties (0.85, [0.77,0.94], *p* = 0.001 and 0.75, [0.70,0.81], *p* < 0.001). Compared with formula feeding, exclusively breastfed children had less communication problems (0.81, [0.74,0.88], *p* = 0.001), social–emotional–behavioural difficulties (0.77, [0.70,0.84], *p* = 0.001), sensory impairments (0.79, [0.65,0.95], *p* = 0.01), physical motor disabilities (0.78, [0.66,0.91], *p* = 0.002), and physical health conditions (0.74, [0.63,0.87], *p* = 0.01). There were no significant associations for mixed-fed children (communication problems (0.94, [0.83,1.06], *p* = 0.312), social–emotional–behavioural difficulties (0.96, [0.85,1.09], *p* = 0.541), sensory impairments (1.07, [0.84,1.37], *p* = 0.579), physical motor disabilities (0.97, [0.78,1.19], *p* = 0.754), and physical health conditions (0.93, [0.74,1.16], *p* = 0.504)). Feeding method was not significantly associated with mental health conditions (exclusive 0.58 [0.33,1.03], *p* = 0.061 and mixed 0.74 [0.36,1.53], *p* = 0.421) or autism (exclusive 0.88 [0.77,1.01], *p* = 0.074 and mixed 1.01 [0.84,1.22], *p* = 0.903). Our study was limited since only 6- to 8-week feeding method was available precluding differentiation between never-breastfed infants and those who stopped breastfeeding before 6 weeks. Additionally, we had no data on maternal and paternal factors such as education level, IQ, employment status, race/ethnicity, or mental and physical health.

**Conclusions:**

In this study, we observed that both breastfeeding and mixed feeding at 6 to 8 weeks were associated with lower risk of all-cause SEN, and SEN attributed to learning disabilities and learning difficulty. Many women struggle to exclusively breastfeed for the full 6 months recommended by WHO; however, this study provides evidence that a shorter duration of nonexclusive breastfeeding could nonetheless be beneficial with regard to the development of SEN. Our findings augment the existing evidence base concerning the advantages of breastfeeding and reinforce the importance of breastfeeding education and support.

## Abstract 2

In a cohort of 191,745 Scottish schoolchildren, Michael Fleming and colleagues explore infant feeding methods and special educational need.

## Author summary

**Why was this study done?:**

- Infant breastfeeding has been associated with reduced physical and mental health problems in childhood which contribute toward special educational need (SEN).
- Previous studies have not investigated the impact of mixed feeding, have not adjusted for child, maternal, and pregnancy confounders, have encountered bias, or have used a narrow definition of SEN.

**What did the researchers do and find?:**

- We linked national, routinely collected, health data (maternity, birth, and health visitor records) and education data (annual school pupil census) together to investigate the association between mode of infant feeding at 6 to 8 weeks of age and both all-cause and cause-specific SEN in Scotland.
- Our study cohort comprised all singleton children, born in Scotland from 2004 onwards with available breastfeeding data and who attended local authority mainstream or special schools in Scotland between 2009 and 2013.
- Outcomes for 48,473 exclusively breastfed children and 16,365 mixed-fed children, respectively, were compared to 126,907 formula-fed children, adjusting for sociodemographic and maternity factors.
- Compared to children who were formula-fed, we observed that, after adjusting for available sociodemographic and maternity factors, exclusively breastfed children and mixed-fed children both had lower risk of all-cause SEN and SEN attributed to learning disabilities and learning difficulties. Exclusively breastfed children additionally had lower risk of communication problems, social–emotional–behavioural difficulties, sensory impairments, physical motor disabilities, and physical health conditions.

**What do these findings mean?:**

- For women who struggle to breastfeed for the full 6 months recommended by WHO, our study suggests that a shorter duration of nonexclusive breastfeeding could still be beneficial with regard to the development of SEN.
- Our findings augment the existing evidence base concerning the advantages of breastfeeding and reinforce the importance of breastfeeding education and support.

## Introduction

The number of children in Scotland with a record of special educational need (SEN) increased almost 4-fold between 2010 and 2018 [1], and, by 2020, almost a third of pupils in Scotland had a record of SEN [2]. While higher case ascertainment may have contributed, it nonetheless represents a significant burden on the education, health, and social sectors, as well as substantial impact on the affected individuals, their families, and wider society [3]. Children with SEN experience lower educational attainment [4,5], higher rates of school absenteeism and exclusion, and higher rates of bullying and maltreatment [6–8], further impacting their physical and mental health and well-being [9]. Discovering modifiable early life risk factors for SEN is therefore important to enable these burdens to potentially be eased through prevention or earlier detection.

Method of infant feeding is one factor that is worth investigating when considering early life risk factors for SEN. It is the recommendation of the World Health Organisation (WHO) that, for the first 6 months of their life, children be breastfed exclusively, due to the health benefits that it affords to both mother and baby [10,11]. Breastfeeding has been shown to be associated with reduced risk of a range of physical health outcomes including gastrointestinal, respiratory, and urinary tract infections, otitis media, asthma, obesity, and diabetes [12,13]. There is also evidence of reduced risk of conditions known to be associated with SEN: autistic spectrum disorder (ASD) [14,15], attention deficit hyperactivity disorder (ADHD) [16,17], communication problems [18,19], mental health problems [20,21], behavioural problems, and impaired social development [22,23], and evidence that breastfeeding may be associated with higher levels of intelligence in later life [24,25]. There is therefore the possibility that breastfeeding could be protective against the development of learning problems, and, given that the most common reason for SEN in Scotland is learning difficulties or disabilities [26], this adds further strength to the hypothesis that infant feeding method may influence the development of all-cause SEN. The nutritional, hormonal, and chemical components of breast milk may directly impact neurodevelopment and risk of disease [27–29]. However, the maternal–infant bonding associated with breastfeeding may also affect child development [30,31].

Previous studies on the associations between infant feeding method and overall risk of outcomes that can contribute to SEN have been limited in number, unable to adjust comprehensively for child, maternal, and pregnancy confounders [32–34], prone to selection bias or recall bias [35,36], unable to take account of mixed feeding [8,32–39], or used a narrow definition of SEN covering only intellectual disability [35]. This study aims to add to the existing evidence and address these limitations by linking national, routinely collected health and educational data together to investigate the association between mode of infant feeding at 6 to 8 weeks of age and both all-cause and cause-specific SEN in Scotland. To the best of our knowledge, the associations between infant feeding method and risk of outcomes contributing to SEN have not previously been investigated on a population-wide scale. Furthermore, this study fills a gap in the literature by investigating a wider range of outcomes than previous studies, investigating formal school-recorded data on SEN, and comparing exclusively breastfed children against mixed-fed children and formula-fed children. We hypothesise that children who are exclusively breastfed will have lower risk of subsequent all-cause and cause-specific SEN compared to children who are mixed-fed and formula-fed.

## Methods

This study is reported as per the Strengthening the Reporting of Observational Studies in Epidemiology (STROBE) guideline (S1 STROBE Checklist). While we did not publish an analysis plan, our analyses were planned before the research team accessed any data. Data were extracted in June 2015, as part of a wider linkage project [40], and this specific study commenced in February 2021, utilising the same data.

### Databases

A Scotland-wide cohort was constructed by linking records, at an individual level, from 3 health (maternity, birth, and health visitor records) and 1 education (annual school pupil census) database. Scottish education and health records contain pupil-unique Scottish Candidate Numbers (SCNs) and patient-unique Community Health Index (CHI) numbers [41], enabling linkage within the specific sectors using deterministic (exact) matching. The health and education data were then linked to each other via probabilistic matching of pupil census education records against the CHI database (population-wide register of all patients in NHS Scotland) using sex, date of birth, and postcode. Linked CHI numbers enabled further deterministic (exact) matching of education records to maternity, birth, and health visitor records. This study formed part of a larger programme of work, and the wider linkage process has been described previously [40,42].

Maternity data were obtained from the Scottish Morbidity Record 02 and the Scottish Birth Record, which record information on obstetric history, the pregnancy and delivery, as well as diagnoses, including congenital anomalies, and immediate outcomes of the offspring. The Scottish Birth Record provides additional information on the offspring up to discharge from hospital, including admission to a special or intensive care unit. The Child Health Pre-School Programme database contains information obtained during the routine child health reviews conducted by health visitors and school nurses [43], including feeding method and developmental progress or delays.

The School Pupil Census covers primary, secondary, and special education and is conducted annually. Completion is a statutory requirement of all local-authority, grant-aided, and special schools in Scotland [26]. The Census gathers aggregated data, such as numbers of pupils and teachers per class, as well as individual-level data including looked after status and SEN [44].

### Inclusion criteria and definitions

Inclusion in the study was restricted to all children who were born in Scotland from 2004 onwards, had complete breastfeeding data, and who attended a Scottish school at some point between 2009 and 2013 inclusive, defined as presence of the pupil on any of the relevant school censuses that are recorded annually every September shortly after the start of the school term. Because of difficulties accurately linking same-sex twins, the study was restricted to singletons. The exposure of interest was infant feeding method ascertained by the health visitor at 6 to 8 weeks of age, and children with missing exposure data were excluded. Feeding method was defined as the predominant method of feeding over the previous 24 hours and classified as (exclusive) breastfeeding, mixed (breastfeeding and formula) feeding, and formula feeding. The outcomes of interest were all-cause SEN and cause-specific SEN. SEN is defined as a requirement for educational arrangements and support above those which are normally provided [26]. SEN was derived from the School Pupil Census, and it is a statutory requirement of Scottish schools to identify, record, and support SEN [26]. Causes of SEN included learning disability, learning difficulty, sensory impairment, physical motor impairment, communication problems, ASD, social–emotional–behavioural difficulties, physical health problems, and mental health problems. These categories are not mutually exclusive; therefore, children can have more than one type of SEN. Children were included in the study regardless of SEN status. Potential confounders included as covariates in the models related to the child (sex, age at School Pupil Census, ethnic group, area-based deprivation quintile at birth), mother (maternal age, smoking status, and marital status), and pregnancy (parity, mode of delivery, sex gestation–specific birth weight centile, gestation at delivery, and 5-minute Apgar score).

### Ethical approval and consent to participate

Approval for the study was obtained from the Public Benefit and Privacy Panel of Public Health Scotland (reference 1920–0144). A data processing agreement was drafted between Glasgow University and Public Health Scotland and a data sharing agreement between Glasgow University and ScotXed. The NHS West of Scotland Research Ethics Service confirmed that formal NHS ethics approval was not required since the study involved anonymised extracts of routinely collected data with an acceptably negligible risk of identification.

### Statistical analyses

The characteristics of participants were summarised by feeding method using frequencies and percentages for categorical data and means and standard deviations for continuous data. The subgroups were compared using Pearson’s χ^2^ test, χ^2^ test for trend, and analysis of variance as relevant. A child could be included in up to 5 School Pupil Censuses. We therefore examined the relationship between infant feeding method at 6 to 8 weeks of age and SEN on a yearly (rather than pupil) basis using generalised estimating equation (GEE) modelling, which took account of serial, correlated outcomes (SEN) relating to the same child across different school years [45]. Analysing the relationship between breastfeeding and SEN on a yearly basis treated each yearly observation as a separate relationship removing the issue of different lengths of follow-up for each child and accounting for changes in the exposure across years. Since these outcomes were binary, the GEE analyses were performed with a binomial distribution and logit link function. The quasi-likelihood under the independence model criterion (QIC) was used to aid the choice of correlation structure [46]. The correlation structure with the lowest QIC value was utilised for all the GEE analyses. Separate models were run for all-cause SEN and each cause-specific SEN. The models were run univariately, partially adjusted (for child confounders) and fully adjusted (for child, maternal, and pregnancy confounders). We tested for statistical interactions between feeding method at 6 to 8 weeks of age, and child age, sex, and area deprivation.

Sensitivity analyses were conducted. The models were rerun excluding children with a congenital anomality, defined as ICD10 codes Q00 to Q99, then excluding children admitted to special care baby units or intensive care units during the first 6 weeks of life. Data cleaning and analyses were conducted using R version 4.2.0 and Stata MP version 17. All analyses were conducted using a *p*-value of <0.05 to determine statistical significance.

## Results

**Fig 1:**
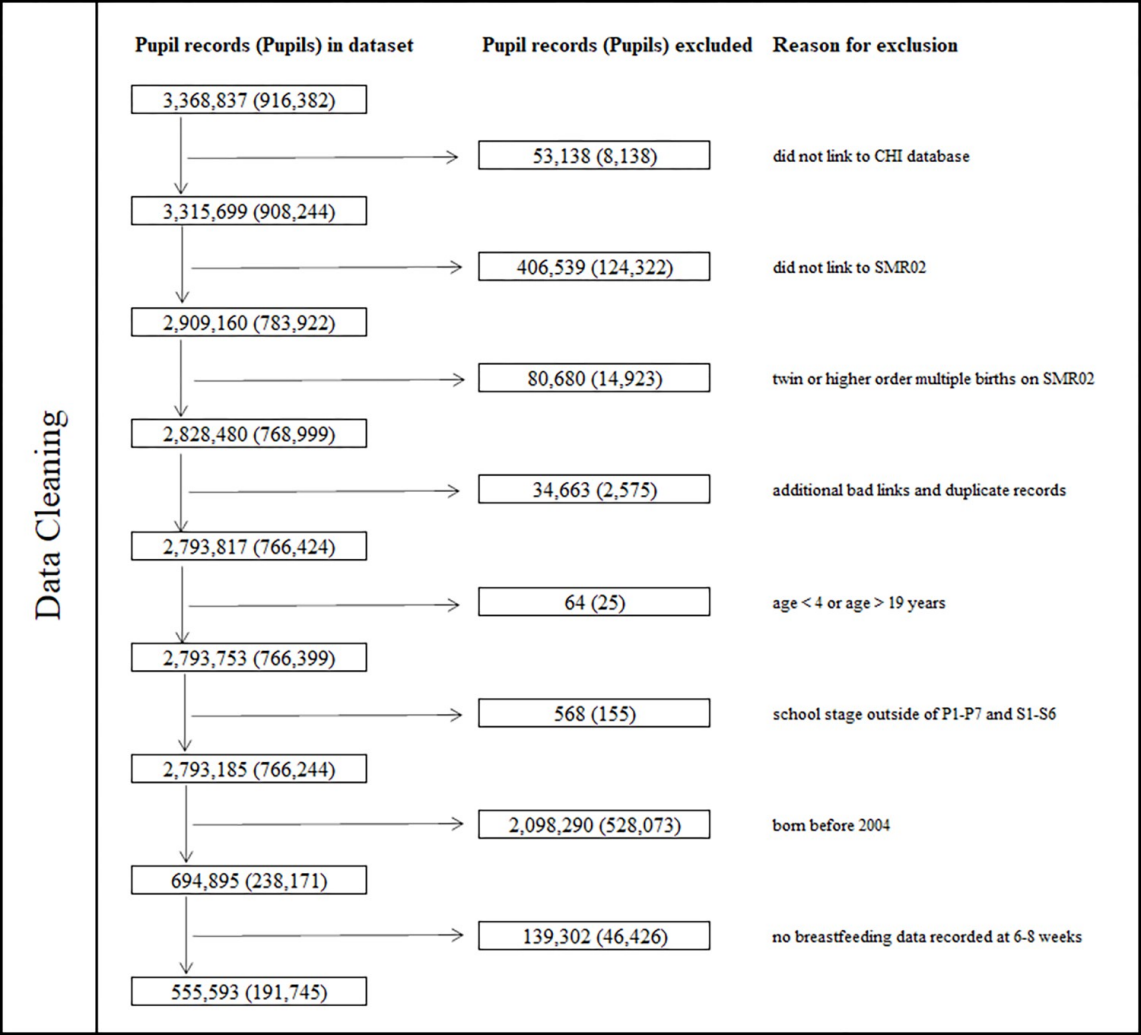
Flow diagram presenting the number of records and pupils excluded at each stage of data cleaning. CHI, Community Health Index; P1–P7 primary 1 through primary 7; SMR, Scottish Morbidity Record; S1–S6, secondary 1 through secondary 6.

The original cohort after data cleaning comprised of 2,793,185 education records pertaining to 766,244 singleton school children born in Scotland and attending school in Scotland between 2009 and 2013. This reduced to 694,875 records pertaining to 238,171 children after limiting to children born on or after 2004. It reduced further to 555,593 records pertaining to 191,745 school children who had complete feeding information recorded at 6 to 8 weeks of age (Fig 1).

Of these 191,745 children, 126,907 (66.2%) were formula-fed, 48,473 (25.3%) were exclusively breastfed, and 16,365 (8.5%) were mixed-fed. All those children, regardless of age, had breastfeeding data, maternity data, and SEN data available. Breastfed children were more likely to be female, lived in more affluent areas, and had mothers who were older, less likely to smoke, and more likely to be married and parous (Table 1). They were less likely to be small for their gestational age, and more likely to be full term at delivery, have had a cephalic vaginal delivery and a 5-minute Apgar score of 7 to 10. The variables with the greatest amount of missing data were marital status (39.93%), smoking during pregnancy (13.99%), and pupil ethnicity (3.45%). However, missing values for these 3 variables were analysed as “unknown” categories and included in all analyses to minimise loss of records. The remaining variables were ordinal categories; therefore, including missing data as unknown categories did not make sense. However, these variables had lower levels of missing values: 5-minute Apgar score (2.81%), child deprivation quintile (2.53%), and parity (1.01%), with the remainder all less than 0.2%. Therefore, we did not deem multiple imputation to be necessary and instead used complete case analyses.

**Table 1:** Child, maternal, and pregnancy characteristics by feeding method at 6–8 weeks of age.

	Formula feeding *N =* 126,907		Mixed feeding *N* = 16,365		Breastfeeding *N* = 48,473		*P* value
	mean	SD	mean	SD	mean	SD	
Age at Census (years)	6.0	0.8	6.0	0.8	6.0	0.8	<0.001^1^
	**N**	**%**	**N**	**%**	**N**	**%**	
Sex							
Male	65,333	51.5	8,598	52.5	23,933	49.4	<0.001^2^
Female	61,574	48.5	7,767	47.5	24,540	50.6	
Deprivation quintile at birth							
1 (most deprived)	45,115	35.6	2,970	18.2	6,898	14.3	<0.001^3^
2	30,542	24.1	3,113	19.1	8,364	17.3	
3	21,481	17.0	3,165	19.4	9,707	20.1	
4	16,767	13.2	3,533	21.6	11,180	23.1	
5 (least deprived)	12,787	10.1	3,556	21.8	12,237	25.3	
Missing	215		28		87		
Ethnic group							
White	118,380	96.7	13,609	86.4	43,140	91.9	<0.001^4^
Asian	2,619	2.1	1,420	9.0	2,118	4.5	
Black	242	0.2	309	2.0	509	1.1	
Other	231	0.2	131	0.8	292	0.6	
Mixed	981	0.8	285	1.8	866	1.8	
Missing	4,454		611		1,548		
Maternal age (years)							
≤24	44,865	35.4	2,361	14.4	5,725	11.8	<0.001^3^
25–29	32,974	26.0	4,003	24.5	11,496	23.7	
30–34	29,727	23.4	5,538	33.8	17,541	36.2	
≥35	19,341	15.2	4,463	27.3	13,711	28.3	
Maternal smoking							
No	74,208	68.8	12,402	87.1	38,914	90.8	<0.001^2^
Yes	33,608	31.2	1,837	12.9	3,955	9.2	
Missing	19,091		2,126		5,604		
Parity							
0	59,361	47.2	7,915	49.0	21,158	44.2	<0.001^3^
1	41,474	33.0	5,272	32.6	16,859	35.2	
>1	24,907	19.8	2,976	18.4	9,877	20.6	
Missing	1,165		202		579		
Marital status							
Married	25,858	34.2	5,636	56.7	18,158	61.5	
Single	43,970	58.1	3,665	36.9	9,542	32.3	<0.001^4^
Other	5,866	7.7	636	6.4	1,848	6.2	
Missing	51,213		6,428		18,925		
Mode of delivery							
Vaginal cephalic	95,504	75.3	11,866	72.5	37,469	77.3	<0.001^4^
Vaginal breech	318	0.3	29	0.2	87	0.2	
Elective CS	12,159	9.6	1,685	10.3	4,303	8.9	
Emergency CS	18,894	14.9	2,778	17.0	6,597	13.6	
Other	32	0.0	7	0.0	17	0.0	
Sex gestation–specific birthweight centile							
1–3	5,794	4.6	551	3.4	1,183	2.4	<0.001^3^
4–10	11,951	9.4	1,289	7.9	3,244	6.7	
11–20	15,458	12.2	1,780	10.9	4,988	10.3	
21–80	73,367	57.8	9,576	58.6	29,622	61.2	
81–90	10,474	8.3	1,631	10.0	4,851	10.0	
91–97	6,613	5.2	1,059	6.5	3,193	6.6	
98–100	3,057	2.4	460	2.8	1,330	2.7	
Missing	193		19		62		
Gestation at delivery (weeks)							
<28	174	0.1	25	0.2	22	0.0	<0.001^3^
28–32	1,240	1.0	128	0.8	152	0.3	
33–36	6,734	5.3	705	4.3	1,483	3.1	
37	6,667	5.3	754	4.6	1,993	4.1	
38	16,225	12.8	1,985	12.1	5,402	11.1	
39	28,008	22.1	3,751	22.9	10,652	22.0	
40	36,096	28.5	4,683	28.6	14,944	30.8	
41	28,198	22.2	3,787	23.2	12,214	25.2	
42	3,333	2.6	525	3.2	1,539	3.2	
>42	158	0.1	14	0.1	54	0.1	
Missing	73		8		18		
5-minute Apgar score							
1–3	443	0.4	60	0.4	131	0.3	<0.001^3^
4–6	1,264	1.0	138	0.9	355	0.8	
7–10	121,665	98.6	15,677	98.8	46,622	99.0	
Missing	3,535		490		1,365		

CS, cesarean section; N, number; SD, standard deviation.

^1^Produced using analysis of variance (ANOVA).

^2^Produced using chi-squared test for trend.

^3^Produced using Spearman rank order correlation.

^4^Produced using chi-squared test for association.

Overall, 23,141 (12.1%) children had a record of SEN: 8,878 (4.6%) had learning difficulties, 6,022 (3.1%) social emotional behavioural difficulties, 5,629 (2.9%) communication problems, 4,389 (2.3%) learning disabilities, 2,137 (1.1%) ASD, 1,749 (0.9%) physical motor impairments, 1,579 (0.8%) physical health problems, 1,223 (0.6%) sensory impairments, and 157 (0.1%) mental health problems. Numbers and percentages of these outcomes by specific feeding type are presented in Table 2.

**Table 2:** Proportion of children with all-cause SEN and cause-specific SEN by feeding method at 6–8 weeks of age.

	Formula feeding		Mixed feeding		Exclusive Breastfeeding		*P* value^1^
	*N* = 126,907		*N* = 16,365		*N* = 48,473		
	*n*	*Col*. *%*	*n*	*Col*. *%*	*n*	*Col*. *%*	
All-Cause SEN							
No	109,492	*86*.*3*	14,750	*90*.*1*	44,362	*91*.*5*	<0.001
Yes	17,415	*13*.*7*	1,615	*9*.*9*	4,111	*8*.*5*	
Cause-Specific SEN: Learning disability							
No	123,446	*97*.*3*	16,092	*98*.*3*	47,818	*98*.*6*	<0.001
Yes	3,461	*2*.*7*	273	*1*.*7*	655	*1*.*4*	
Cause-Specific SEN: Learning difficulty							
No	120,145	*94*.*7*	15,785	*96*.*5*	46,937	*96*.*8*	<0.001
Yes	6,762	*5*.*3*	580	*3*.*5*	1,536	*3*.*2*	
Cause-Specific SEN: Sensory impairment							
No	126,013	*99*.*3*	16,260	*99*.*4*	48,249	*99*.*5*	<0.001
Yes	894	*0*.*7*	105	*0*.*6*	224	*0*.*5*	
Cause-Specific SEN: Physical motor impairment							
No	125,638	*99*.*0*	16,218	*99*.*1*	48,140	*99*.*3*	<0.001
Yes	1,269	*1*.*0*	147	*0*.*9*	333	*0*.*7*	
Cause-Specific SEN: Communication problems							
No	122,757	*96*.*7*	15,947	*97*.*4*	47,412	*97*.*8*	<0.001
Yes	4,150	*3*.*3*	418	*2*.*6*	1,061	*2*.*2*	
Cause-Specific SEN: ASD							
No	125,398	*98*.*8*	16,179	*98*.*9*	48,031	*99*.*1*	<0.001
Yes	1,509	*1*.*2*	186	*1*.*1*	442	*0*.*9*	
Cause-Specific SEN: Social–emotional–behavioural difficulties							
No	122,142	*96*.*2*	15,981	*97*.*7*	47,600	*98*.*2*	<0.001
Yes	4,765	*3*.*8*	384	*2*.*3*	873	*1*.*8*	
Cause-Specific SEN: Physical health problem							
No	125,723	*99*.*1*	16,245	*99*.*3*	48,198	*99*.*4*	<0.001
Yes	1,184	*0*.*9*	120	*0*.*7*	275	*0*.*6*	
Cause-Specific SEN: Mental health problem							
No	126,785	*99*.*9*	16,354	*99*.*9*	48,449	*100*.*0*	0.002
Yes	122	*0*.*1*	11	*0*.*1*	24	*0*.*0*	

ASD, autism spectrum disorder; Col % column percentage; SEN, special educational need.

^1^Produced using chi-squared test for trend.

Compared with children who were formula-fed, we observed that children who were mixed-fed (OR 0.70, 95% CI [0.66,0.74], *p* < 0.001) and children who were exclusively breastfed (OR 0.59 (95% CI [0.57,0.61], *p* < 0.001) were both less likely to have all-cause SEN on univariate analysis. Following adjustment for child, maternal, and pregnancy confounders, the associations were attenuated but remained significant (mixed feeding OR 0.90, 95% CI [0.84,0.95], *p* < 0.001 and exclusive breast-feeding OR 0.78, 95% CI [0.75,0.82], *p* < 0001) (Table 3).

Compared with children who were formula-fed, we observed that children who were mixed-fed or were exclusively breastfed were less likely to have learning disabilities (mixed feeding OR 0.75, 95% CI [0.65,0.87], *p* < 0.001 and exclusive breastfeeding OR 0.66, 95% CI [0.59,0.74], *p* < 0.001), or learning difficulties (mixed feeding OR 0.85, 95% CI [0.77,0.94], *p* = 0.001 and exclusive breastfeeding OR 0.75, 95% CI [0.70,0.81], *p* < 0.001). Compared with children who were formula-fed, we observed that children who were exclusively breastfed were less likely to have communication problems (OR 0.81, 95% CI [0.74,0.88], *p* < 0.001), social–emotional–behavioural difficulties (OR 0.77, 95% CI [0.70,0.84], *p* < 0.001), sensory impairments (OR 0.79, 95% CI [0.65,0.95], *p* = 0.01), physical motor disabilities (OR 0.78, 95% CI [0.66,0.91], *p* = 0.002), and physical health conditions (OR 0.74, 95% CI [0.63,0.87], *p* < 0.001) after adjusting for potential confounders. Feeding method was not significantly associated with ASD or mental health conditions (Table 3).

No statistically significant interactions between feeding method and either pupil sex, pupil age, or pupil socioeconomic status were identified. Furthermore, exclusion of the 7,533 children who had a congenital abnormality and exclusion of the 13,193 children who were admitted to intensive care or special care baby units did not alter the findings (S1 Table).

**Table 3:** Univariate and multivariate associations between infant feeding method at 6–8 weeks of age and all-cause and cause-specific SEN.

	Univariate *N* = 191,745 children (555,593 records)			Adjusted for child confounders *N* = 191,415 children (554,621 records)			Adjusted for child, maternal and pregnancy confounders *N* = 183,881 children (528,554 records)		
	OR	95% CI	*p*-value	OR	95% CI	*p*-value	OR	95% CI	*p*-value
All-cause SEN									
Formula	1.00			1.00			1.00		
Mixed	0.70	0.66–0.74	<0.001	0.79	0.74–0.84	<0.001	0.90	0.84–0.95	<0.001
Breast	0.59	0.57–0.61	<0.001	0.68	0.66–0.71	<0.001	0.78	0.75–0.82	<0.001
Learning disability									
Formula	1.00			1.00			1.00		
Mixed	0.62	0.54–0.71	<0.001	0.68	0.59–0.78	<0.001	0.75	0.65–0.87	<0.001
Breast	0.50	0.46–0.55	<0.001	0.59	0.53–0.65	<0.001	0.66	0.59–0.74	<0.001
Learning difficulty									
Formula	1.00			1.00			1.00		
Mixed	0.65	0.59–0.72	<0.001	0.74	0.67–0.82	<0.001	0.85	0.77–0.94	0.001
Breast	0.57	0.54–0.61	<0.001	0.65	0.61–0.70	<0.001	0.75	0.70–0.81	<0.001
Communication problems									
Formula	1.00			1.00			1.00		
Mixed	0.82	0.73–0.92	<0.001	0.85	0.76–0.96	0.008	0.94	0.83–1.06	0.312
Breast	0.66	0.61–0.71	<0.001	0.73	0.67–0.79	<0.001	0.81	0.74–0.88	<0.001
Social–emotional–behavioural difficulties									
Formula	1.00			1.00			1.00		
Mixed	0.61	0.54–0.69	<0.001	0.77	0.68–0.88	<0.001	0.96	0.85–1.09	0.541
Breast	0.46	0.42–0.50	<0.001	0.61	0.56–0.66	<0.001	0.77	0.70–0.84	<0.001
Autistic spectrum disorder									
Formula	1.00			1.00			1.00		
Mixed	1.01	0.85–1.21	0.870	1.05	0.88–1.26	0.581	1.01	0.84–1.22	0.903
Breast	0.83	0.74–0.94	0.003	0.93	0.82–1.06	0.291	0.88	0.77–1.01	0.074
Sensory impairment									
Formula	1.00			1.00			1.00		
Mixed	0.94	0.75–1.19	0.601	0.97	0.77–1.23	0.816	1.07	0.84–1.37	0.579
Breast	0.63	0.54–0.75	<0.001	0.66	0.56–0.79	<0.001	0.79	0.65–0.95	0.010
Physical motor disability									
Formula	1.00			1.00			1.00		
Mixed	0.86	0.71–1.05	0.144	0.91	0.74–1.11	0.350	0.97	0.78–1.19	0.754
Breast	0.68	0.60–0.79	<0.001	0.72	0.62–0.83	<0.001	0.78	0.66–0.91	0.002
Physical health condition									
Formula	1.00			1.00			1.00		
Mixed	0.84	0.68–1.04	0.109	0.92	0.74–1.14	0.445	0.93	0.74–1.16	0.504
Breast	0.63	0.54–0.73	<0.001	0.70	0.60–0.81	<0.001	0.74	0.63–0.87	<0.001
Mental health condition									
Formula	1.00			1.00			1.00		
Mixed	0.51	0.26–1.02	0.056	0.61	0.31–1.21	0.156	0.74	0.36–1.53	0.421
Breast	0.40	0.25–0.65	<0.001	0.47	0.29–0.77	0.002	0.58	0.33–1.03	0.061

CI, confidence interval; OR, odds ratio; SEN, special educational need.

Adjusted for child (sex, age at school pupil census, ethnic group, area-based deprivation quintile at birth), mother (maternal age, smoking status, and marital status), and pregnancy (parity, mode of delivery, sex gestation–specific birth weight centile, gestation at delivery, and 5-minute Apgar score) factors.

## Discussion

In this study, we observed that compared to children who were formula-fed, exclusively breastfed children had less risk of all-cause SEN and SEN attributed to learning disabilities, learning difficulties, communication problems, social–emotional–behavioural difficulties, sensory impairments, physical motor disabilities, and physical health conditions after adjusting for available sociodemographic and maternity factors. Compared to children who were formula-fed, those who were mixed-fed also appeared to be less likely to have all cause SEN and SEN attributed to learning disabilities or learning difficulties.

Our findings concur with a previous case–control study in which 49 individuals aged 13 to 22 years who were receiving additional learning support for presumed cognitive difficulties were less likely to have been breastfed on discharge from hospital than their siblings and an unrelated control group [35]. Our finding that breastfeeding was associated with less communication problems is consistent with the findings in previous studies [18,19]. Social–emotional–behavioural problems have also been examined previously, but with both exclusive and nonexclusive breastfeeding being found to be protective, in contrast to our findings [17,22]. Surprisingly, our study found no significant relationship between either breastfeeding or mixed feeding and ASD. This conflicts with two previous meta-analyses that both reported that breastfeeding was protective against ASD [14,15]. This may be due to our study including less severe cases in which the association may be weaker or nonexistent.

Congenital anomalies can impact on infant feeding choices [47–49], as well as predisposing to SEN [36,50]. Similarly, admission to intensive care and special care baby units can impact feeding choice [51–53] and is associated with SEN [35]. Many previous studies have included children with congenital anomalies or those admitted into neonatal units but not adjusted for these as potential confounders [8,32,33,36,38,39]. In our study, we observed that the association between breastfeeding and reduced risk of SEN was present among children who did not have congenital anomalies and among children not admitted to intensive care or special care baby units. However, a limitation of our study was lack of data on specific lengths of stay in intensive care or special care baby units.

Children who were born in private hospitals, privately educated, or homeschooled were not included in the study. However, only 4% of children in Scotland attend an independent school [54], and homeschooling and private maternity care are very uncommon [55]. Because of the need to link birth and education records, we could not include children who were not born in Scotland or who emigrated from Scotland before starting school.

Previous studies on breastfeeding and SEN have analysed smaller study populations [35,36] and been prone to selection bias [8,38], recall bias [8,33,34,36,38], and loss to follow-up. Use of national routine data enabled us to conduct a large-scale, unselective study that included hard-to-reach groups. Since both exposure and outcome data were collected routinely by the health and education sectors, recall bias was avoided.

Many previous studies have analysed feeding method as a binary variable [32,33,35]. We were additionally able to investigate children who were mixed-fed. While self-reported feeding method was based on predominant feeding method in the preceding 24 hours and was therefore not affected by recall bias, it was not corroborated and therefore could not differentiate infants who had never been breastfed from those in whom it had been discontinued prior to 6 weeks. Scottish data show that rates of breastfeeding drop off between first visit, 6- to 8-week review, and 13- to 15-month review [56]. Therefore, status at 6 to 8 weeks gives a snapshot picture but cannot be applied to wider time points. We also did not have information on the type of formula feed, whether any children received donor milk, or use of expressed breast milk. The latter is important since skin-to-skin contact increases maternal bonding and is beneficial for cognition [30,31].

Health visitors are funded by the National Health Service, which is free to the whole population at the point of delivery. The Scottish Government’s core programme of child health reviews and screening activities are a universal process that are offered to every child. Beyond core contacts, the health visitor uses professional judgement to decide on further contacts. While there is no legal requirement to have a child health review, and it is not mandatory that mothers accept a child health visit, recent data from Public Health Scotland show that 97% of children successfully receive their first child review (in the days after birth) and 90% successfully receive their 6- to 8-week review with uptake similar across different ethnic groups, maternal ages, and deprivation categories. [57] In our study, based on older data, around 20% of pupil records had no 6- to 8-week review data; however, this figure was similar regardless of mother’s maternal age, or pupil’s ethnicity or deprivation, suggesting that missing data on more vulnerable groups of children is unlikely. Indeed, the percentage of missing data was least among most deprived children and children of Asian ethnicity.

The risk of incomplete or inaccurate data inherent in secondary data analysis was mitigated by the recording of SEN being a statutory requirement. Prevalence of SEN increased over the duration of the study from 2% to 12% and has continued to increase in recent years. This increase can be largely attributed to increased ascertainment of conditions such as ASD and ADHD due to greater awareness among parents and teachers. Since the observed increase was similar irrespective of exposure group, it is unlikely to have introduced bias. We also adjusted all of our analyses for pupil age. The requirement for SEN is decided by the teacher, and the school’s special educational needs coordinator (SENCO) in discussion with the child’s parents or guardians, taking account of formal and informal assessments and wider information about progress and desired outcomes. The additional support put in place can vary significantly and is dependent on the individual child’s requirements [3]. The categories for the cause-specific SENs are completed within the education sector, and the person deciding on what is recorded can vary from teacher to medical professional to support staff such as an educational psychologist. It is possible that some children with needs will not meet the threshold to be formally offered support while others may have difficulties that are not picked up. The study was retrospective and used administrative data. Therefore, it is not possible to verify that ascertainment of SEN was 100% complete as this would require prospective recruitment and examination/investigation of all children. However, likelihood of complete ascertainment is increased due to the benefits of formal diagnoses, the legal requirements to identify and record SEN, and the fact that ascertainment can be triggered by teachers, parents, and healthcare workers. Furthermore, there is no reason to believe that ascertainment is likely to differ systematically by breastfeeding history, and, therefore, that bias was introduced.

The results of this study were obtained based on Scottish data. Rates of breastfeeding, however, vary greatly across the United Kingdom, Europe, and worldwide. It has been reported that overall prevalence of breastfeeding varies from 30.7% in high-income countries to over 90% in low- and middle-income countries [58]. This makes it difficult to generalize our findings to low- and middle-income countries. Our observed breastfeeding rate of 33.8% (either exclusive or mixed breastfeeding) agrees broadly with rates reported across high-income countries.

We had access to several potential confounders not available in some previous studies [8,35–37,39], but, as with any observational study, residual confounding is possible. We did not have any data on important maternal or paternal factors such as education level [59,60], intelligence quotient (IQ) [61], employment status, race/ethnicity, or mental and physical health; however, we did adjust for the mother’s area socioeconomic status at the time of birth, which is a proxy measure. One of the most frequently seen confounders within the literature is parental education or intelligence level. Children born to mothers or fathers who are more educated or have a higher IQ appear to have lower likelihood of the most common cause of SEN—learning difficulties [59,60,62]. There is also evidence that parents who have reached higher education levels are more likely to breastfeed [63,64]. Other sociodemographic factors may also act as confounders in the relationship. Older mothers, those from Black and Minority ethnic groups, those who are married or cohabiting, and those in higher socioeconomic classes have been reported to be more likely to breastfeed [63,65]. Some of these characteristics are also associated with intellectual problems. For example, it has been stated that divorced or single mothers are more likely to have children with cognitive delay [66]; children from families with a higher socioeconomic position are more likely to do better academically [60,67]; and, among late and moderately preterm children, non-white ethnicity is a significant risk factor for cognitive impairment [35]. Our data agreed with previous literature with respect to breastfeeding rates being lower among younger and more deprived mothers. The reasons for these patterns should be explored in more detail.

There are several areas where further research would be beneficial. Firstly, being able to examine the impact of duration of different feeding types on all-cause and cause-specific SEN in a large population cohort such as this would provide insights into any dose–response relationships that may be present, and whether there are any critical feeding durations. Furthermore, research regarding expressed breast milk versus breastfeeding on a large representative sample would enhance understanding of whether it is the act of breastfeeding, or breast milk itself that impacts upon SEN. Finally, with regard to the exposure, it would be useful to know whether the relationship between feeding and SEN is maintained for different types of formula feed, as well as donor milk compared with mother’s milk.

Residual confounding could be responsible for the observed associations in this study, and so performing the same methodology on a large sample with a greater number of confounders is also recommended, particularly including confounders that were not included in this study due to lack of data such as maternal IQ, parental education level and occupation, and alcohol or drug use during pregnancy.

Future studies are also required to investigate whether the impact of neonatal stays on the relationship between mixed feeding and SEN applies for all lengths of neonatal stay. Unfortunately, this was not possible to conduct within this piece of research due to lack of available data around length of stay. Finally, there has been some research that suggests that breastfeeding is linked to reduced hospitalisation and reduced chronic disease in childhood [12,68–70]. Certain chronic diseases have also been associated with SEN [71–74]. There is the possibility, therefore, that childhood chronic disease acts as a mediator between feeding method in infancy and SEN, and so further research is required to examine this relationship.

In conclusion, breastfeeding may be protective against children developing SEN. While exclusive breastfeeding is the ideal, even mixed feeding may confer some benefit. The results of this study suggest that feeding method in infancy could be a modifiable risk factor for all-cause SEN, which has the potential to help reduce its burden in relation to the affected children, their families, and wider society. We observed that, compared to formula-fed children, children who were breastfed and children who were mixed-fed at 6 to 8 weeks had lower risk of all-cause SEN, and SEN attributed to learning disabilities and learning difficulty. This is significant as many women struggle to exclusively breastfeed for the full 6 months recommended by WHO [10] for a host of different reasons [11]. However, this study provides evidence that even a short duration of nonexclusive breastfeeding could be beneficial with regard to development of SEN. Our findings augment the existing evidence base concerning the advantages of breastfeeding and reinforce the importance of breastfeeding education and support. Several meta-analyses have been conducted examining the effectiveness of breastfeeding education and support programmes, and these all concluded that the programmes were beneficial as regards increasing the initiation and duration of breastfeeding [75,76]. Despite breastfeeding rates increasing in recent years, the rates in Scotland vary by maternal age, socioeconomic status, and education level and are lower than rates in England [56]. Policies such as Scotland’s 2019 published recommendations to make the country breastfeeding friendly [77] should therefore remain at the forefront of political and public health agendas.

## Supporting information

S1 Table Multivariate associations between infant feeding method at 6–8 weeks of age and all-cause and cause-specific special educational need after excluding children with a congenital abnormality and children who were admitted to intensive or special care units. ASD, autistic spectrum disorder; Col. %, column percentage; OR, odds ratio. Adjusted for child (sex, age at school pupil census, ethnic group, area-based deprivation quintile at birth), mother (maternal age, smoking status, and marital status), and pregnancy (parity, mode of delivery, sex gestation–specific birth weight centile, gestation at delivery, and 5-minute Apgar score) factors.
(DOCX)

S1 STROBE Checklist STROBE Statement—Checklist of items that should be included in reports of observational studies. (DOCX)

## Abbreviations

ADHD: attention deficit hyperactivity disorder
ASD: autistic spectrum disorder
CHI: Community Health Index
GEE: generalised estimating equation
IQ: intelligence quotient
QIC: quasi-likelihood under the independence model criterion
SCN: Scottish Candidate Number
SEN: special educational need
SENCO: special educational needs coordinator
WHO: World Health Organisation
