# Supplementary material for: Infant feeding method and special educational need in 191,745 Scottish schoolchildren: A national, population cohort study
Source: PLoS Med. 2023 Apr 6;20(4):e1004191. doi: 10.1371/journal.pmed.1004191 (PMC10079126; doi:10.1371/journal.pmed.1004191)
Supplement: S1 Table — ASD, autistic spectrum disorder; Col. %, column percentage; OR, odds ratio. Adjusted for child (sex, age at school pupil census, ethnic group, area-based deprivation quintile at birth), mother (maternal age, smoking status, and marital status), and pregnancy (parity, mode of delivery, sex gestation–specific birth weight centile, gestation at delivery, and 5-minute Apgar score) factors. (DOCX) [file pmed.1004191.s001.docx]

**Supplementary Table 1** Multivariate associations between infant feeding method at 6-8 weeks of age and all-cause and cause-specific special educational need after excluding children with a congenital abnormality and children who were admitted to intensive or special care units

|  | **Multivariate Analysis after excluding children with a congenital abnormality** | | | **Multivariate Analysis after excluding children admitted to intensive or special care units** | | |
| --- | --- | --- | --- | --- | --- | --- |
|  | **OR** | ***95% CI*** | **p value** | **OR** | ***95% CI*** | **p value** |
| **All Cause SEN** |  |  |  |  |  |  |
| Formula feeding | 1.00 |  |  | 1.00 |  |  |
| Mixed feeding | 0.90 | 0.84-0.95 | <0.001 | 0.90 | 0.84-0.96 | 0.002 |
| Exclusive breast feeding | 0.78 | 0.75-0.82 | <0.001 | 0.80 | 0.77-0.84 | <0.001 |
| **Learning Disability** |  |  |  |  |  |  |
| Formula feeding | 1.00 |  |  | 1.00 |  |  |
| Mixed feeding | 0.75 | 0.65-0.87 | <0.001 | 0.76 | 0.65-0.89 | <0.001 |
| Exclusive breast feeding | 0.66 | 0.59-0.74 | <0.001 | 0.70 | 0.63-0.78 | <0.001 |
| **Learning Difficulty** |  |  |  |  |  |  |
| Formula feeding | 1.00 |  |  | 1.00 |  |  |
| Mixed feeding | 0.85 | 0.77-0.94 | 0.001 | 0.86 | 0.77-0.95 | 0.005 |
| Exclusive breast feeding | 0.75 | 0.70-0.81 | <0.001 | 0.77 | 0.71-0.82 | <0.001 |
| **Communication Problems** |  |  |  |  |  |  |
| Formula feeding | 1.00 |  |  | 1.00 |  |  |
| Mixed feeding | 0.94 | 0.83-1.06 | 0.312 | 0.95 | 0.83-1.08 | 0.425 |
| Exclusive breast feeding | 0.81 | 0.74-0.88 | <0.001 | 0.84 | 0.77-0.92 | <0.001 |
| **ASD** |  |  |  |  |  |  |
| Formula feeding | 1.00 |  |  | 1.00 |  |  |
| Mixed feeding | 1.01 | 0.84-1.22 | 0.903 | 0.98 | 0.80-1.19 | 0.822 |
| Exclusive breast feeding | 0.88 | 0.77-1.01 | 0.074 | 0.88 | 0.76-1.02 | 0.088 |
| **Social-emotional-behavioural difficulties** |  |  |  |  |  |  |
| Formula feeding | 1.00 |  |  | 1.00 |  |  |
| Mixed feeding | 0.96 | 0.85-1.09 | 0.541 | 0.95 | 0.83-1.08 | 0.443 |
| Exclusive breast feeding | 0.77 | 0.70-0.84 | <0.001 | 0.77 | 0.70-0.85 | <0.001 |
| **Sensory Impairment** |  |  |  |  |  |  |
| Formula feeding | 1.00 |  |  | 1.00 |  |  |
| Mixed feeding | 1.07 | 0.84-1.37 | 0.579 | 1.05 | 0.80-1.38 | 0.722 |
| Exclusive breast feeding | 0.79 | 0.65-0.95 | 0.010 | 0.86 | 0.70-1.04 | 0.123 |
| **Physical Motor Disability** |  |  |  |  |  |  |
| Formula feeding | 1.00 |  |  | 1.00 |  |  |
| Mixed feeding | 0.97 | 0.78-1.19 | 0.754 | 0.91 | 0.72-1.15 | 0.426 |
| Exclusive breast feeding | 0.78 | 0.66-0.91 | 0.002 | 0.78 | 0.66-0.93 | 0.006 |
| **Physical Health Condition** |  |  |  |  |  |  |
| Formula feeding | 1.00 |  |  | 1.00 |  |  |
| Mixed feeding | 0.93 | 0.74-1.16 | 0.504 | 0.96 | 0.76-1.22 | 0.747 |
| Exclusive breast feeding | 0.74 | 0.63-0.87 | <0.001 | 0.76 | 0.64-0.91 | 0.003 |
| **Mental Health Condition** |  |  |  |  |  |  |
| Formula feeding | 1.00 |  |  | 1.00 |  |  |
| Mixed feeding | 0.74 | 0.36-1.53 | 0.421 | 0.63 | 0.32-1.26 | 0.190 |
| Exclusive breast feeding | 0.58 | 0.33-1.03 | 0.061 | 0.62 | 0.34-1.11 | 0.107 |
|  | |  | |  | |  |
| Col. % column percentage; OR Odds Ratio; ASD Autistic Spectrum Disorder  Adjusted for child (sex, age at school pupil census, ethnic group, area-based deprivation quintile at birth), mother (maternal age, smoking status, and marital status), and pregnancy (parity, mode of delivery, sex-gestation-specific birth weight centile, gestation at delivery and 5-minute Apgar score) factors | | | | | | |
|  | | | | | | |
